# Achieving Successful Extubation and Cost-Effective Recovery Following Anesthetic Airway Management in Supracarinal Tracheal Reconstruction Surgeries: A Retrospective Analysis

**DOI:** 10.7759/cureus.34225

**Published:** 2023-01-26

**Authors:** Amuktamalyada Mulakaluri, Pateel GNP, Subramanya Rao P, Babu MS, Rathna Bai Nanjunda Rao

**Affiliations:** 1 Department of Anaesthesiology, Rangadore Memorial hospital, Bangalore, IND; 2 Department of Anaesthesiology, Rangadore Memorial Hospital, Bangalore, IND; 3 Department of Otolaryngology-Oncology, Rangadore Memorial Hospital, Bangalore, IND; 4 Department of Otolaryngology-Laryngology, Rangadore Memorial Hospital, Bangalore, IND

**Keywords:** apnoea ventilation apnoea, cross field ventilation, distal tracheal intubation, anaesthetic considerations, airway management, tracheal reconstruction

## Abstract

Introduction

From an anesthesiologist’s perspective, perioperative concerns related to supracarinal tracheal reconstruction surgery include having uninterrupted smooth ventilation without any laryngeal edema, glottic dysfunction, and airway leak. Surgical concerns comprise various kinds of anastomotic dissections, fistulas to innominate arteries, and the esophagus. The most serious complication following tracheal surgery is anastomotic separation, which might manifest modestly as stridor, respiratory distress, and extremis. To avoid dire repercussions, prompt management and securing the airway are necessary. Against this background, we wanted to highlight the importance of early extubation and discharge of supracarinal tracheal reconstruction patients from hospitals without any postoperative complications and with the least expenses possible, since most of these patients have already undergone postintubation tracheal stenosis and prolonged intensive care unit stay, and have experienced significant financial burden incurring from preceding events.

Methodology

Medical records of all patients admitted for tracheal reconstruction during the period from March 2019 to April 2022 (four years) were reviewed to collect patient demographic details, surgical descriptions, anesthesia data, records of pre-anesthetic evaluations, and postoperative details up until the hospital discharge.

Results

The most common reason for tracheal stenosis among our patients was post-intubation tracheal stenosis (PITS), which was seen in 8/13 patients (61.53%); 4/13 patients (30.76%) had stridor at rest and underwent emergency tracheostomy preoperatively immediately following admission to the hospital. The stenosis was situated at a median distance of 3 cm [interquartile range (IQR): 0.5-7] from the true vocal cords or 7 cm (IQR: 3-9) from the carina. The median length of tracheal resection was 2 cm (IQR: 1-4). We observed that the mode of induction for airway management was tracheostomy tube in four patients (with 90% tracheal stenosis), placement of laryngeal mask airway (LMA) with spontaneous ventilation in four patients (with 75% tracheal stenosis), and small-size (#5-7.5 sizes) endotracheal tube (ETT) placement in five patients (with less than 75% tracheal stenosis). The postoperative complication noted was bleeding from the operative site in 1/13 patients (7.6%); a 0% mortality rate was noted during the hospital stay and up until six months post-discharge. We noted that the median duration of postoperative hospitalization was five days (IQR: 2-15), and the total cost incurred by each patient was less than INR 85,000 (USD 1,000).

Conclusion

Our analysis revealed that all our patients were extubated in the operative room and shifted to the ward. In the "open airway phase", standard distal tracheal intubation and cross-field ventilation techniques, and tracheal suturing were facilitated by the apnoea-ventilation-apnoea technique. Both the techniques along with the emergency tracheostomies done in severe tracheal obstruction preoperatively and intraoperative anesthesia management with the insertion of LMA Supreme, maintained with spontaneous breathing techniques, offered potential advantages in the management of supracarinal tracheal reconstruction surgeries. The multidisciplinary teamwork along with close communication and good rapport with the surgical team was found to be the key factor in the fast-track extubation and recovery of these patients.

## Introduction

Background

In 1896, Koenig performed the first recorded tracheal repair on an individual, grafting connective tissue and a piece of rib over a tracheal fistula in a two-step procedure [1]. Given the trachea's poor blood supply, continual direct exposure of the upper airway to the atmosphere, and complicated mechanical expectations of the tissue, long-segment tracheal defects - typically defined as segments longer than 5 cm in adults and relatively short segments in children - are particularly challenging to treat. Bruns performed the first lengthy annular reconstruction of the trachea in 1898 [2]. In the past 45 years, tracheal surgery has advanced to the point where it is now possible to resect around half of the adult trachea with primary reconstruction, mostly by anatomic mobilizing methods. Resection and rebuilding techniques for the laryngotracheal and carina have also been established with a high degree of reliability [3].

As described by Sandberg and Warren, in the anesthetic management of tracheal reconstruction surgeries, three out of five stages are critical, which are as follows: airway management during induction and intubation, cross-field or jet ventilation during an "open airway" phase, and extubation and postoperative care [4]. The procedure is often carried out under general anesthesia that involves intermittent tailored doses of both intravenous and inhalational anesthetic agents or only total intravenous anesthesia (TIVA) [5]. The procedure most frequently involves inserting a small-size endotracheal tube (ETT) close to the stenosis, the use of a laryngeal mask airway (LMA) for supracarinal tracheal resection and reconstruction (TRR), and catheter-based high-frequency jet ventilation techniques for airway management [6].

Among all the patients who underwent tracheal reconstruction surgeries at our tertiary care center, the major cause of tracheal stenosis was post-intubation tracheal stenosis (PITS) [7,8]. PITS patients were referred from different hospitals with various etiological factors during the pre-coronavirus disease 2019 (COVID-19) and post-COVID-19 periods. These patients had tracheal stenosis due to prolonged intubation, and underwent a subsequent tracheostomy during the preceding event, causing further harm to the already impaired tracheal lumen [9,10].

Impact statement

Conventionally, tracheal reconstruction surgeries involve prolonged hospitalization, intraoperative invasive monitoring, sophisticated equipment, unique ventilation strategies, and postoperative intensive care monitoring, and they are costly procedures [11], especially for people in countries with low socioeconomic indicators. With the aim of prioritizing care to such patients, supracarinal tracheal reconstruction surgeries have been performed at our institution with conventional, most reliable ventilation techniques like distal tracheal intubation with cross-field ventilation strategies [12,13], managed intraoperatively with standard anesthesia monitoring, and the patients' condition is monitored with utmost vigilance, both preoperatively, with a view to properly preparing them, and postoperatively to maintain the ventilation and oxygenation. We found that this has ensured all our (supracarinal) tracheal reconstruction surgical patients had a minimal length of hospital stay and incurred the least cost possible. All our patients have been successfully extubated in the operating room and shifted to the ward.

Due to the compromised airway and the requirement to share the airway with the surgeon while maintaining the respiratory function, anesthesia for TRR is very challenging [14]. The preoperative evaluation and strategy for induction in the presence of tracheal pathology, the collaboration with the surgeon during airway excision and anastomosis, emergence, and postoperative care are all unique to this procedure [15]. The type, location, and morphology of the obstructive mass, as well as the severity and grading of airway obstruction [16-18] and surgical technique, influence airway management methods [12].

While the earlier studies have focused on the techniques used during the procedure, they did not delve into the cost and duration of hospitalization. Consequently, in previous studies, patients had an expensive and longer duration of hospital stay [11]. Our study focused on identifying factors that reduced the cost and duration of hospitalization.

Study objectives

We conducted a retrospective study to analyze our experiences with 13 patients who underwent TRR surgeries utilizing various airway management procedures [12,13] at our institution during the four-year period from 2019 to 2022.

The primary and secondary objectives of our study were as follows: to analyze the management and formulate strategies for different airway management techniques, depending upon the severity of obstruction and tracheal diameter during upper airway surgeries. The various techniques used were intubation with direct laryngoscopy/awake fiberoptic intubation, LMA placement [5], placement of a small-size ETT [4], or using a tracheostomy tube placed preoperatively as an emergency airway in severe tracheal stenosis patients presenting with stridor at rest [19]. Spontaneous ventilation techniques for patients with critical airway obstruction [20-22] as well as postoperative outcomes and complications were reviewed and the methods to reduce the cost and duration of hospitalization were identified.

## Materials and methods

Study design, setting, and data collection

This retrospective study was approved by the Rangadore Memorial Hospital Ethics Committee (approval no: RMHEC/AL/05/2022). Medical records of all the patients admitted for tracheal reconstruction surgeries at a single tertiary hospital during the period from March 2019 to April 2022 were reviewed. All patients aged 15-85 years who underwent tracheal reconstruction surgeries during this four-year period were included in the study. We reviewed the surgical descriptions, anesthetic records, pre-anesthetic evaluations, and details up to the hospital discharge. Medical, nursing, and respiratory treatment data were also examined for the following details.

Preoperative Period

Patient demographic data, preoperative patient condition, reasons for tracheal stenosis, comorbidities, room-air oxygen saturation (SpO_2_), vitals, presence of functional tracheostomy, details of emergency tracheostomy done, and patients’ airway and pulmonary condition based on CT neck scan-3D reconstruction were collected.

Intraoperative Data

Fiberoptic bronchoscopy (FOB) findings for the type and location of the stenosis/lesion, the duration of anesthesia (minutes), duration of surgery (minutes), lowest oxygen saturation (SpO_2_%), peak end-tidal carbon dioxide (ETCO_2_ in mmHg), and high peak airway pressures (HPP) were noted.

Postoperative Data

Postoperatively, data related to any complications, total duration of hospital stay, duration of ICU stay, and the total cost incurred by the patient were calculated.

**Figure 1:**
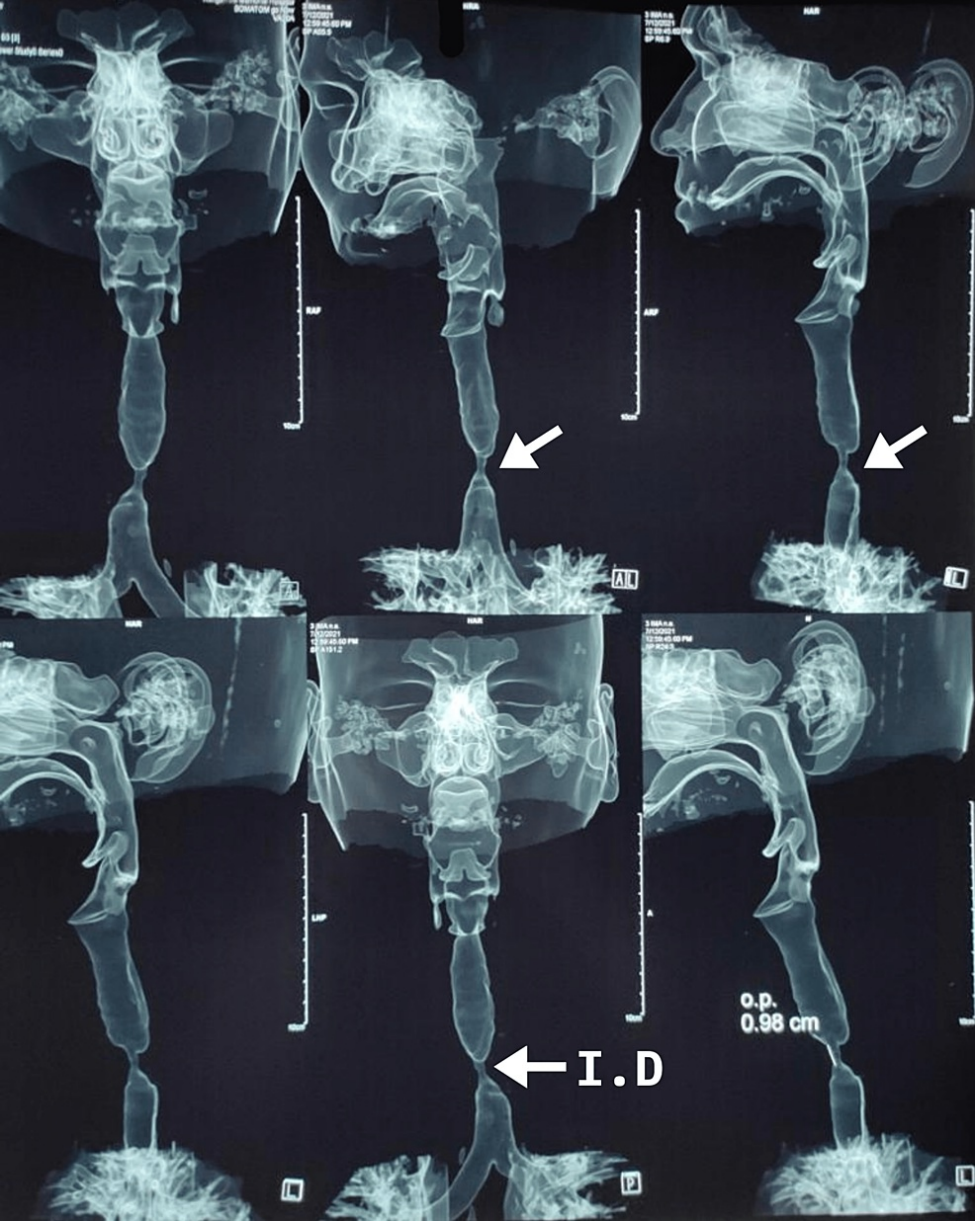
CT image of patient no. 7 showing the tracheal stenosis Tracheal stenosis was noted at the tracheal rings T2-T3 (illustrated by white arrows). Internal diameter (I.D.) at the level of stenosis was 6 mm; outer diameter (O.D.) at the level of stenosis was 0.98 cm. The patient was intubated orally with an endotracheal tube 7.5 fixed at 18 cm, placed above the obstruction CT: computed tomography

Data were collected and analyzed retrospectively. The anesthetic induction was planned depending on preoperative CT neck-3D reconstruction of the trachea (Figure 1) and intraoperative pre-induction FOB (Video 1) findings of the nature, growth pattern, size, position, degree of airway blockage, length, and proximity of narrowing from either the vocal cords or carina [2].

**Video 1:** Fiberoptic endoscopy for tracheal reconstruction Fiberoptic bronchoscopy was done to analyze the type and location of tracheal stenosis intraoperatively for a patient with post-intubation tracheal stenosis

Patients were classified into three categories based on the severity of the obstruction, and the severity of tracheal stenosis (depending upon preop CT/awake FOB findings - pre-induction); accordingly, three different modes of airway management were adopted, which were as follows:

1. If the tracheal obstruction involved 90% of the tracheal lumen (as found in four patients: patients 1, 2, 4, and 11), emergency tracheostomy was done preoperatively immediately after admission and patients were brought to the operating room with a tracheostomy tube and induced with the same. This planning proved to be highly beneficial in terms of patient safety and the management of the most severe tracheal obstruction during the induction of general anesthesia.

2. If the tracheal obstruction was more than 75% (as found in four patients: patients 8, 9,10, and 12), intravenous (IV) premedication, induction with titrated doses of the injection propofol, fentanyl IV, and LMA placement with spontaneous ventilation were preferred, due to the fibrotic scar tissue in the stenotic area, with the anticipated difficulty in trans-stenotic ETT placement.

3. If the obstruction was less than 75% (as found in five patients: patients 3, 5, 6, 7, and 13) with an unobstructed tracheal diameter of more than 5 mm, the placement of small-size (#5-7.5 sizes) ETT was preferred.

In patients 3, 5, and 6, #5-size ETT was placed, which could ensure sufficient trans-stenotic space to intubate. As the tube was placed beyond the stenosed segment with the help of FOB, the tube was fixed at depths ranging from 18 to 20 cm, which was variable in each patient according to the level of tracheal stenosis. In patient no. 7, #7.5 ETT was placed and fixed at 18 cm at the corner of the mouth to be above the T2-T3 tracheal stenosis with 6-mm luminal narrowing. The highest peak airway pressure observed was 25 cmH_2_O in this patient. In patient no. 13, who had carcinoma of the thyroid, tumor extension into the right vocal cord and anterior commissure was suspected, with subglottic T2-T3 tracheal narrowing 60% (6 mm), because of which awake fiberoptic nasotracheal intubation was performed with 7-size Flexometallic ETT; the tube was fixed at a depth of 22 cm at the nasal level. In all these patients, muscle relaxant was given after the confirmation of the placement of ETT for controlled ventilation.

Anesthetic management in detail

For all non-tracheostomized patients, the airway was prepared for FOB with 4% lidocaine 4-ml nebulization, and nasal packing with 2% lignocaine and adrenaline-soaked gauze before the induction of anesthesia, for the placement of either small-size ETT or LMA. During FOB, oxygen was administered by either using the working channel connected to a continuous oxygen delivery tube technique or the apneic ventilation technique depending on the severity of the tracheal obstruction.

If LMA (LMA Supreme™ Second Generation) was placed (due to the reasons described above), premedication with intravenous injections of glycopyrrolate 0.2 mg, midazolam 1 mg, spontaneous ventilation with IV propofol induction with titrated doses of 0.5-1 mg/kg body weight for insertion of the LMA, followed by infusion for TIVA dose titrated to 0.3-2 mg/kg body weight was administered until the tracheal wall was incised [5].

General anesthesia was induced for all other patients using IV injections of glycopyrrolate 0.2 mg, midazolam 1 mg, and propofol 1-2 mg/kg in combination with fentanyl 2μg/kg in incremental doses after the ventilation was confirmed successfully with ETCO_2_ capnography vial ventilating with face-mask oxygen of 6 liters per minute and sevoflurane 1% v/v. Confirmation of correct placement of small-size ETT with FOB visualization or tracheostomy tube, with ETCO_2_ and bilateral equal air entry by auscultation, was mandatory before the administration of the muscle relaxant IV atracurium (0.5 mg/kg body weight) for the initiation of controlled ventilation.

Patients were monitored with standard anesthesia monitoring involving multiparameter monitoring with oxygen saturation, ETCO_2_, heart rate, noninvasive blood pressure, electrocardiography, and ventilator monitoring with all parameters of pressure/volume control ventilation, tidal volume, respiratory rate, and peak airway pressure.

**Figure 2:**
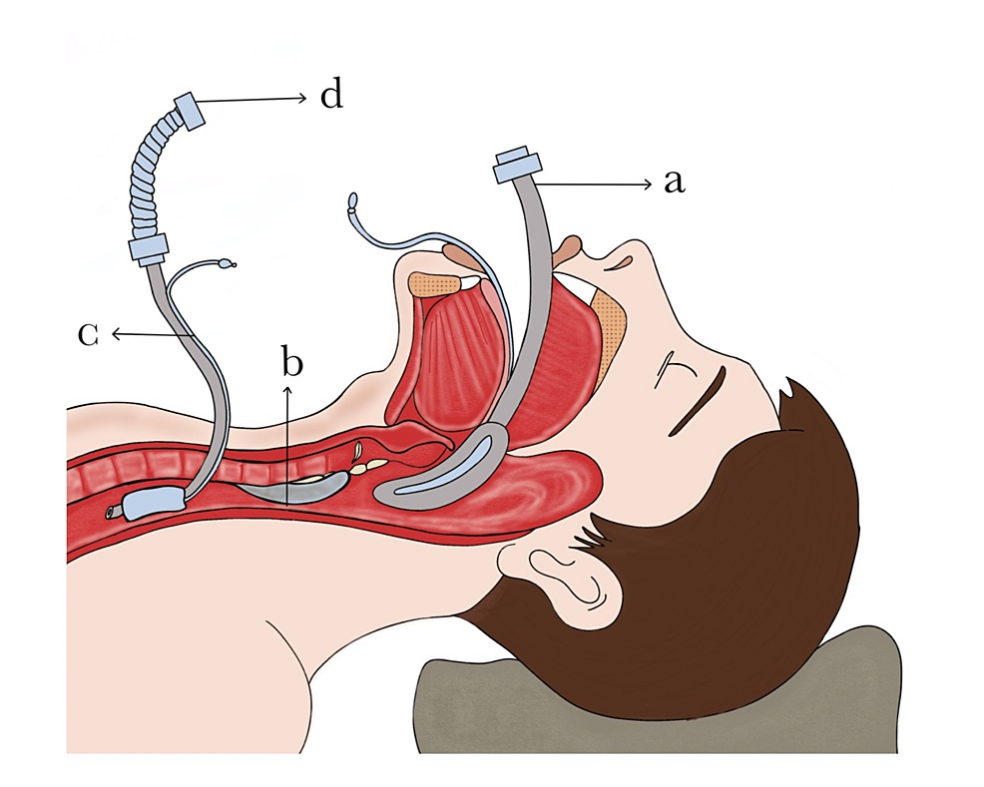
Placement of laryngeal mask airway for tracheal stenosis of more than 75% a. Induction with laryngeal mask airway. b. Tracheal stenosis of more than 75%. c. Distal intubation with Flexometallic intratracheal tube during open airway. d. Cross-field ventilation during tracheal resection Original diagram by Dr. Amuktamalyada M

During the open-airway phase of our study, distal tracheal intubation was done by surgeons with sterile ETT of appropriate size into the distal trachea, which was connected to a sterile catheter mount and sterile circle system circuit wrapped in a sterile plastic cover connected to an anesthesia machine for cross-field ventilation [5,13]. Tracheal suturing was facilitated by the apnoea-ventilation-apnoea technique (Figure 2) [12]. Once the diseased trachea was removed and the posterior wall of the trachea was sutured, the new appropriate-size orotracheal tube was inserted beyond the anastomotic site under direct vision with surgical guidance.

Following the completion of the sutures, the surgeons verified that there were no air leaks from the anastomotic site, and the anesthesiologist confirmed the same with a cuff leak test. If a negative cuff leak was found, the ventilation parameters were also checked. As per the extubation protocol, alert, conscious, and spontaneously breathing patients with good tidal volume with normocapnia were extubated, only after achieving hemodynamic stability and restoration of adequate airway reflexes.

Statistical analysis

Statistical analyses were performed using IBM SPSS Statistics version 25.0 (IBM Corp., Armonk, NY) for windows. The findings were reported as median, interquartile range (IQR), absolute numbers (n), and percentages.

## Results

Demographics and other data of the patients are presented in Table 1. Among patients who underwent tracheal resection, 76.9% were middle-aged males aged more than 32 years. The lowest peripheral oxygen saturation observed was 88% (in patient 1) and was graded as American Society of Anesthesiologists (ASA) 1V. The most common symptom before surgery among our patients was exertion dyspnoea (n=10) and stridor at rest (n=4). Due to preoperative respiratory distress, 4/13 patients underwent emergency tracheostomy and were stabilized before surgery (Table 1).

**Table 1:** Baseline demographic and clinical characteristics of patients (n=13) ASA: American Society of Anesthesiologists; IQR: interquartile range; SpO_2_: peripheral oxygen saturation

Characteristics	Values
Age, years, median (IQR)	33 (18–63)
Weight, kg, median (IQR)	65 (50–120)
Preoperative SpO_2_, median (IQR)	96 (88–120)
Sex, n (%)	
Male	10 (76.92%)
Female	3 (23.07%)
ASA physical status, n (%)	
II	8 (61.53%)
III	4 (30.76%)
IV	1 (7.69%)
Preoperative exertion dyspnea, n (%)	10 (76.92%)
Preoperative stridor at rest, n (%)	4 (30.76%)
Preoperative respiratory failure, n (%)	3 (23.07%)

The most prevalent causes of tracheal stenosis were prolonged mechanical ventilation (PITS, n=8), followed by primary malignant tracheal tumors (n=2), an extension of tumors of the thyroid into the trachea (n=2), and idiopathic origin (n=1). The stenosis was situated at a median distance of 3 cm (IQR: 0.5-7) from the vocal cords and 7 cm (IQR: 3-9) from the carina. The median length of tracheal resection was 2 cm (IQR: 1-4) (Table 2).

**Table 2:** Characteristics of obstructive mass and approaches for airway management Small-size (5) ETT: small-sized endotracheal tube sized 5; LMA: laryngeal mask airway

Characteristics	Values
Distance from the vocal cord, cm, median (IQR)	3 (0.5–7)
Distance from carina, cm, median (IQR)	7 (3–9)
Tracheal stenosis resection length, cm, median (IQR)	2 (1–4)
Tracheal stenosis level, %, median (IQR)	90 (60–90)
Causes of tracheal obstruction, n	
Primary tracheal tumor	2
Thyroid carcinoma extension involving the trachea	2
Post-intubation/prolonged ventilation	8
Idiopathic	1
Approach for airway management, n	
Tracheostomy tube induction	4
Small-size (5) ETT tube	3
Non-intubation LMA	4
Fiberoptic nasotracheal intubation	1
Oral intubation	1

Among eight patients (61.53%) with post-intubation tracheal stenosis, the various causes for prolonged intubation-ventilation were as follows: head injury due to a road traffic accident and assault in two patients, post-COVID-19 PITS in one patient, post-COVID-19 recovery-mucormycosis in one patient for which extensive facial-maxillary debridement was done, organophosphorus poisoning in one patient, urosepsis/acute kidney failure in two patients, and respiratory failure due to pulmonary consolidation and pulmonary tuberculosis in one patient.

The median time for anesthesia was 180 minutes (IQR: 90-495) and the median operation time was 180 minutes (IQR: 80-480). Among all the patients intraoperatively, the lowest SpO_2_ was 92% (in patients 3 and 12); the highest ETCO_2_ was 60 mmHg in patient 3 and above 55 mmHg in patients 8,9, and 10. The maximum blood loss noted of 1300 ml occurred only in one patient (patient 13) with carcinoma of the thyroid. Tumor extension into the right vocal cord and anterior commissure was suspected in this patient, with subglottic T2-T3 tracheal narrowing of 60% (6-mm tracheal lumen); the tracheal segment resected was 3 cm long with extensive neck dissection for complete margin clearance from the tumor. Two packed RBCs were transfused intraoperatively for this patient (Table 3). This patient had bleeding from the suture line on postoperative day two, for which he was shifted to ICU for re-suturing under local anesthesia.

**Table 3:** Intraoperative data ETT: endotracheal tube; FLEX: Flexometallic (reinforced) endotracheal tube; SpO_2_: peripheral oxygen saturation; ETCO_2_: end-tidal carbon dioxide; HPP: high peak airway pressure measured; MAP: mean arterial pressure; TV: tidal volume; HR: heart rate; LMA: laryngeal mask airway; T-tube: tracheostomy tube; FOB: fiberoptic bronchoscope intubation

Patient no.	Airway management during induction	Tracheal resection: intratracheal ETT	Post-tracheal reconstruction oral intubation	Duration of surgery (minutes)	Duration of anesthesia (minutes)	Duration of ventilation without muscle relaxation (minutes)	Lowest SpO_2_ (%)	Highest ETCo_2_ (mmHg)	HPP (cmH_2_O)	TV (ml)	MAP (mmHg)	HR (bpm)
1.	T-tube	6.5 FLEX	6.5 FLEX	180	195	Nil	96	34	18	500	123	70
2.	T-tube	8 FLEX	8 FLEX	480	495	Nil	96	35	18	500	93	78
3.	5 ETT	TRC-8 PORTEX	TRC-8 PORTEX	80	90	45	92	60	38	500	90	78
4.	T-tube	7 FLEX	7 FLEX	150	165	Nil	100	39	18	500	90	110
5.	5 ETT	5 ETT	5 ETT	165	180	Nil	96	44	28	450	100	88
6.	5 ETT	7 FLEX	7 FLEX	180	180	60	98	42	28	500	93	80
7.	7.5 ETT - fixed at 18 cm	7 FLEX	6.5 FLEX	240	240	40	99	46	25	500	100	92
8.	LMA 4	7.5 FLEX	7.5 FLEX	165	180	35	97	55	44	350	80	110
9.	LMA 3	7 FLEX	7 FLEX	150	165	30	96	58	48	344	76.6	98
10.	LMA 3	6.5 FLEX	6.5 FLEX	120	135	30	98	56	46	285	80	78
11.	T-tube	6.5 FLEX	6.5 FLEX	180	180	Nil	99	39	20	500	106.6	100
12.	LMA 3	6 FLEX	6 FLEX	225	240	60	92	48	48	500	123	90
13.	FOB nasal int 7.5 ETT	7 FLEX	7 FLEX	300	300	Nil	99	36	38	500	133	80
Median				180	180	-	97	44	28	500	93	88
Range				80–480	90–495	30–60	92–100	34–60	18–48	350–500	76.6–133	70–110

In our study, all the patients were extubated on the table in the operating room. The patients were subsequently managed in the ward. Postoperatively, two patients were shifted to ICU. The median postoperative hospitalization period in the ward was five days (IQR: 2-15). In-hospital mortality encountered was 0% (Table 4). Patient 2 had papillary thyroid carcinoma infiltrated exclusively into the tracheal tissues. This patient had tracheal stenosis subglottic level (5 mm from true vocal cords); the tracheal segment resected was the longest (4 cm), involving cricoid and T1-T3 tracheal rings, followed by unique longitudinal tracheal reconstruction using costal cartilage. The frozen sections were sent from the operating room for confirmation of the dissected margins. The surgical (480 minutes) and anesthesia (495 minutes) duration were the longest in this patient compared to others. This patient had the longest duration of hospital stay (15 days) due to the complexity of the surgery and was discharged only after complete recovery postoperatively (as his remote hometown 120 km away did not have standard healthcare facilities) (Table 4).

**Table 4:** Postoperative data ETT: endotracheal tube; OT: operation theatre; ICU: intensive care unit; INR: Indian rupees, USD: United States dollar

Patient no.	Extubation of the ETT inside OR	ICU	Duration of ICU stay (days)	Duration of hospital stay (days)	Hospital expenses (INR)	Hospital expenses (USD)	Postoperative complications
1	Y	Nil	0	5	65,000.0	802.62	Tracheostomy performed after 1 month
2	Y	Nil	0	15	75,000.0	926.11	None
3	Y	Nil	0	2	20,000.0	246.96	None
4	Y	Nil	0	4	40,000.0	493.92	None
5	Y	Nil	0	5	60,000.0	740.88	None
6	Y	Nil	0	5	60,000.0	740.88	None
7	Y	Post extubation	2	9	80,000.0	987.85	None
8	Y	Nil	0	6	60,000.0	740.88	None
9	Y	Nil	0	2	20,000.0	246.96	None
10	Y	Nil	0	3	40,000.0	493.92	None
11	Y	Nil	0	4	60,000.0	740.88	None
12	Y	Nil	0	4	60,000.0	740.88	None
13	Y	Postop day 2	3	9	80,000.0	987.85	Post-thyroidectomy bleeding on postop day 2

## Discussion

In our study, postoperative bleeding was noted in only one patient; our study revealed a 7.6% complication rate and a 0% hospital mortality rate up until six months post-discharge. All our patients were preoperatively educated about the "guardian stitch" and the importance of maintaining neck flexion. Post-extubation in the operative room, the patients were shifted to the ward with proper postoperative instructions given to ward staff. Only 2/13 patients were admitted to the ICU after extubation: patient 7 with sternotomy and intercostal drainage tube from the operating room, alert and conscious spontaneously breathing without any respiratory exertion for postoperative monitoring and treatment; and patient 13 who had carcinoma thyroid that had extended to tracheal rings T2 and T3 and had extensive neck dissection, who was shifted to ICU from the ward on postoperative day two due to bleeding from the surgical site that required resealing sutures.

The study by Liu et al. [11] showed a mortality rate of 15.38%, and prolonged ICU stays (5-12 days), which contrasts with our study where the postop ward stay was 2-15 days, which resulted in significant cost reduction. The minimum total cost incurred by any patient in the Liu study was more than 6,000 USD [11], which is significantly higher than in our study where the total cost incurred was less than 1,000 USD. Dedicated institutes that perform TRR have achieved >90% successful outcomes, with a low mortality rate of 1-2%. Even in these facilities, the patients were monitored in the ICU postoperatively [23]. The cost differs significantly between developed and developing countries, starting from hospital admissions, bed charges, and consumables; the primary determining factor for "the cost" was whether the patients were operated on using sophisticated instruments and sent to an ICU after surgery for ventilation, invasive monitoring, and daily investigations.

Wright et al. conducted a retrospective analysis of 901 TRR cases, which revealed a complication rate of 18.2%. Their study extensively focused on postoperative complications such as an anastomotic leak and factors that help in the prediction of these high-risk patients [19]. Similarly, in our study, the signs that will manifest in case of anastomotic dehiscence, like tachypnoea, noisy breathing (stridor), and respiratory distress, were explained to our patients and ward staff, which decreased both cost and complications in our study.

Marwaha et al. provided an overview of the classification of tracheal stenosis, various anesthetic management strategies available, decision-making related to airway control, and novel techniques. Their study involved 70 patients and it revealed a 4% complication rate and a 1% mortality rate [12]. Ranganath et al. focused on thyroid cancer cases and the involvement of the trachea, in about one-third of locally advanced cancers. Their study was conducted on 10 patients and showed a 30% complication rate and a 10% mortality rate [13]. Both these studies have mentioned the use of cross-field ventilation and apnoea-ventilation-apnoea technique during tracheal suturing, which were employed in our supracarinal reconstruction surgeries.

The most common reason for tracheal stenosis observed was prolonged mechanical ventilation and tracheostomy (PITS) [24]. The patients who had PITS had various etiologies, such as head injury, urosepsis, organic phosphorous poisoning, post-tracheostomy following extensive facial-maxillary surgery, and post-COVID-19 recovery [7,8].

The clinical assessment was found to be the key component of airway management [11]. When the tracheal diameter is reduced to 50% or it approaches a value of 8 mm, exertion dyspnea develops [25,26]. When the inner diameter was less than 5-6 mm, the patient had stridor even at rest [27]; 3D reconstruction images of the trachea (CT neck) and FOB performed intraoperatively to assess the nature, growth pattern, size, position, degree of airway blockage, length, and proximity of narrowing from the true vocal cords and carina [12] were found to be the key factors for successful airway management and recovery.

As described earlier, If more than 90% of the tracheal lumen was blocked, an emergency tracheostomy was performed preoperatively, and these patients came to the operating room with a functional tracheostomy tube and were induced with the same. If the tracheal obstruction was more than 75%, LMA placement with spontaneous ventilation was preferred [28]. If the obstruction was less than 75%, with an unobstructed tracheal diameter of more than 5 mm and soft or intratracheal mass not prone to bleeding or detachment, placement of small-size ETT was preferred [29].

The patient’s airway was prepared for FOB, with nebulization and airway topicalization. It was quite effective in managing spontaneous ventilation without inducing cough during the open airway phase when the patient was maintained completely on total intravenous anesthesia [12]. Patients were monitored with standard anesthesia monitoring [30] as described earlier, which proved sufficient for safe anesthesia and successful recovery of our patients.

As for the comparison of all three airway strategies used in our study, HPP and peak ETCO_2_ during the pre-tracheal resection phase were observed in patients with LMA Supreme Second Generation on spontaneous ventilation for a short duration until the definitive intratracheal ETT was secured. According to a previous case report by Huang et al. (2017), the indications for non-intubated spontaneous respiration anesthesia are as follows: (I) no contraindications for anesthesia or surgery; (II) no severe cardiopulmonary diseases; and (III) low airway secretions. Likewise, in our study, no complications were observed in patients with a spontaneous ventilation approach [20].

**Figure 3:**
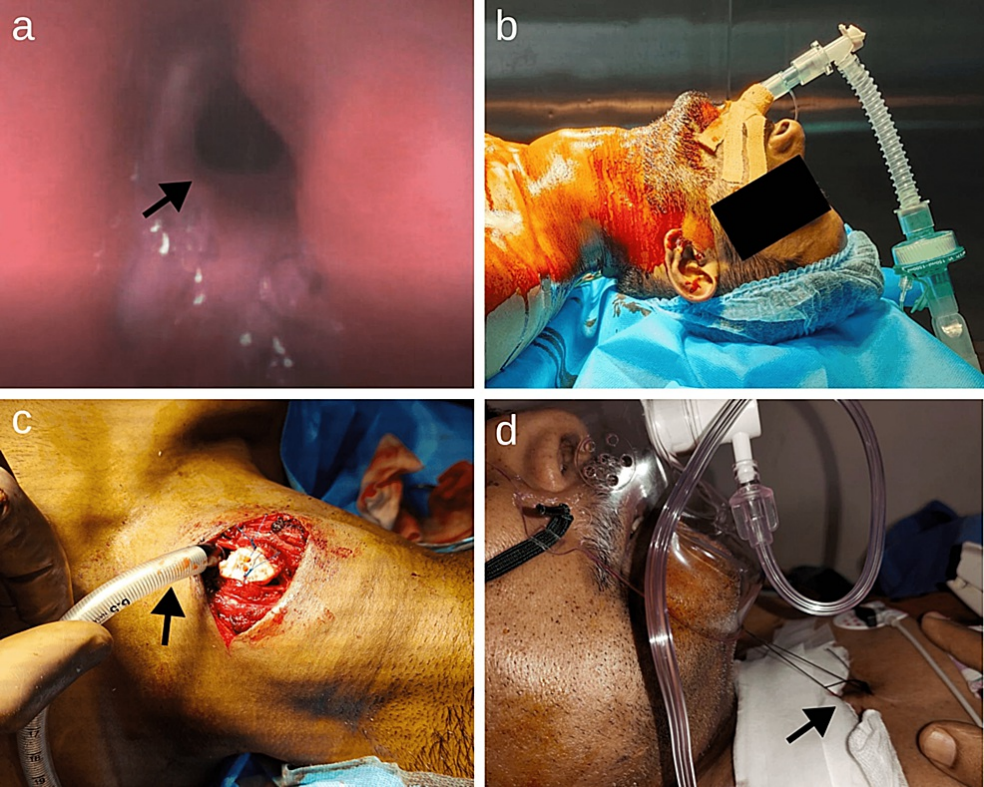
Tracheal stenosis of more than 75%: induction with laryngeal mask airway a. Tracheal stenosis of more than 75%. b. Induction with laryngeal mask airway. c. Distal tracheal intubation and cross-field ventilation. d. Placement of guardian suture (illustrated by black arrows) to avoid the stretch of tracheal anastomosis

During the transient period of the tracheal resection due to the leak, low tidal volume with acceptable hypercapnia and low SpO_2_ were noted, which were stabilized as soon as the intratracheal tube was secured and ventilation resumed. Although the patients presented transient hypoxia and hypercapnia during the pre-tracheal resection phase, all our patients had rapid recovery from the anesthesia. This was attributed to the adequate anesthetic depth, monitoring, timing, and administration of muscle relaxants (Figure 3).

Open airway phase

**Figure 4:**
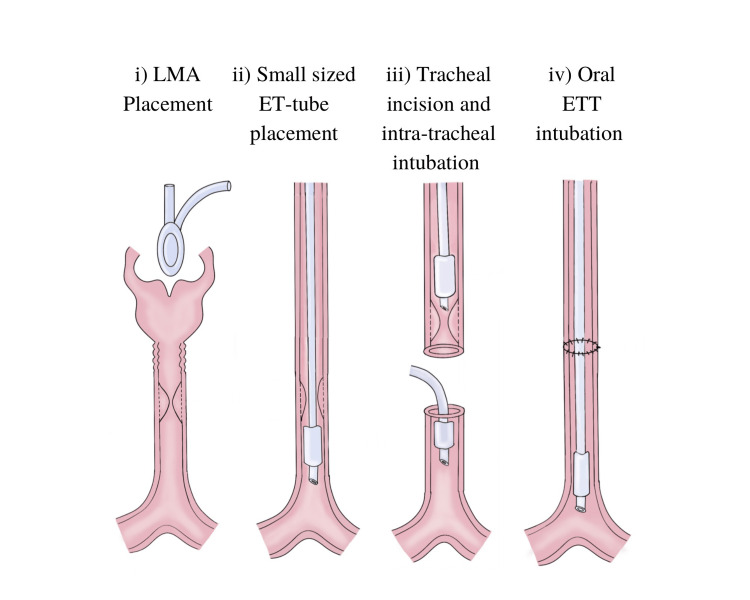
The diagrammatic representation of the three-way procedure adapted for tracheal reconstruction Placement of laryngeal mask airway for tracheal stenosis of more than 75%, small-size endotracheal tube for less than 75% stenosis, withdrawal of this tube during open airway phase, distal tracheal intubation, and cross-field ventilation (intratracheal intubation), and, finally, the placement of oral endotracheal tube and advancement through the anastomosis under direct vision LMA: laryngeal mask airway; ETT: endotracheal tube Original diagram by Dr. Amuktamalyada M

Propofol, fentanyl, and atracurium were administered intravenously to maintain a sufficient depth of anesthesia, especially when the tracheal wall was incised. Inhalational agents were stopped. Intravenous propofol (TIVA) and oxygen were administered until the intratracheal ETT was placed distal to the obstruction through which cross-field ventilation was resumed. Tracheal suturing was facilitated by the apnoea-ventilation-apnoea technique. After the diseased trachea was removed and the posterior wall of the trachea was sutured, the new appropriate-size orotracheal tube was inserted beyond the anastomotic site under direct vision. The diagrammatic representation of the three-way procedure we adapted for tracheal reconstruction is shown in Figure 4.

Postoperatively, comprehensive ancillary care consisting of humidified oxygen through a face mask, nebulization, antitussives, gentle physiotherapy, and pulmonary toileting was administered to the patients in the ward under monitoring. Patients were kept in a propped-up position up to >45 degrees, with side supports to the neck to avoid neck rotation and extension, and were explained the purpose of "guardian stitch". Patients were also educated to avoid straining, coughing, and forceful movements that increase the stress on tracheal sutures. This differs from the previous studies where patients were admitted to ICU and managed with invasive/periodic blood gas monitoring during the first 24 hours [12,13]. Similarly, our patients and ward staff were educated to look for postoperative tachypnoea, dyspnoea, respiratory distress, cough, noisy breathing (stridor), bleeding from the operative site, fever, and desaturation signs that will manifest in case of anastomotic dehiscence. These postoperative measures in our study decreased both cost and complications. Patients were discharged only after complete recovery. The postoperative follow-up was done weekly for the first two months, followed by every two weeks for the next six months, by the surgical team.

Limitations of the study

Our study had several drawbacks. Primarily, our sample size was small, which could have prevented us from drawing firm conclusions about the advantages and drawbacks of the three airway management regimens we followed. Secondly, as this was a retrospective analysis, no post-discharge information was gathered, including clinical symptoms, improvements in the quality of life, and long-term survival. Moreover, the study was limited to patients who had supracarinal surgeries and did not include much more challenging surgeries like carinal and subcarinal surgeries involving open/video-assisted thoracotomies. This was a single-arm study without a control group and was confined to a single institute. Due to the financial constraints of the patients, we performed standard anesthesia monitoring only, with close personal monitoring in the postoperative wards.

## Conclusions

Based on our experience with the 13 patients described in this study, the anesthetic airway management strategy should be chosen based on the condition of the individual patient, the preference of the patient, and airway pathology. The emergency tracheostomies done in severe tracheal obstruction preoperatively, intraoperative anesthesia management with the insertion of LMA, maintained with spontaneous ventilation techniques, and the standard distal tracheal intubation and cross-field ventilation techniques employed in the “open airway phase”, offered potential advantages in the management of our supracarinal tracheal reconstruction surgeries. Multidisciplinary teamwork, close communication, and good rapport with the surgical team built over four years, along with the nursing team cooperation, were key factors in the proper management of these patients preoperatively, intraoperatively, and postoperatively in the ward. And thanks to these factors, we were able to reduce the duration of hospital stays and patient expenses significantly.
